# The Chondro-Osseous Continuum: Is It Possible to Unlock the Potential Assigned Within?

**DOI:** 10.3389/fbioe.2018.00028

**Published:** 2018-03-21

**Authors:** Behzad Javaheri, Soraia P. Caetano-Silva, Ioannis Kanakis, George Bou-Gharios, Andrew A. Pitsillides

**Affiliations:** ^1^Skeletal Biology Group, Comparative Biomedical Sciences, The Royal Veterinary College, London, United Kingdom; ^2^Institute of Ageing and Chronic Disease, University of Liverpool, Liverpool, United Kingdom

**Keywords:** chondrocyte, transdifferentiation, cartilage, bone, extracellular matrix, osteoblast

## Abstract

Endochondral ossification (EO), by which long bones of the axial skeleton form, is a tightly regulated process involving chondrocyte maturation with successive stages of proliferation, maturation, and hypertrophy, accompanied by cartilage matrix synthesis, calcification, and angiogenesis, followed by osteoblast-mediated ossification. This developmental sequence reappears during fracture repair and in osteoarthritic etiopathology. These similarities suggest that EO, and the cells involved, are of great clinical importance for bone regeneration as it could provide novel targeted approaches to increase specific signaling to promote fracture healing, and if regulated appropriately in the treatment of osteoarthritis. The long-held accepted dogma states that hypertrophic chondrocytes are terminally differentiated and will eventually undergo apoptosis. In this mini review, we will explore recent evidence from experiments that revisit the idea that hypertrophic chondrocytes have pluripotent capacity and may instead transdifferentiate into a specific sub-population of osteoblast cells. There are multiple lines of evidence, including our own, showing that local, selective alterations in cartilage extracellular matrix (ECM) remodeling also indelibly alter bone quality. This would be consistent with the hypothesis that osteoblast behavior in long bones is regulated by a combination of their lineage origins and the epigenetic effects of chondrocyte-derived ECM which they encounter during their recruitment. Further exploration of these processes could help to unlock potential novel targets for bone repair and regeneration and in the treatment of osteoarthritis.

## Introduction

The mobility provided by a robust locomotor skeletal system is a major determinant of human health and quality of life. Loss of mobility is a leading cause of ill-health and ultimately death. Two common skeletal problems include defective bone healing and osteoarthritis (OA); despite increasing incidence their treatment remains unmodified for many years (Woolf and Pfleger, 2003; Hunter et al., 2014). Their scale is huge; some 1:25 people suffers a bone fracture each year and prevalence in the aged reaches 50%, yet as many as 20% do not heal (Johnell and Kanis, 2005). Large bone defects resulting from tumor resection add to this population. In these patients, sites from which bone is harvested often require follow-up surgery. The greatest mobility failure, however, arises from OA a painful, disabling syndrome affecting one third of those >65 years and many younger people. Despite its extensive impact, OA treatment options remain palliative and conclude most often in joint replacement. These two problems are inextricably linked by cartilage and bone pathology, repair, and development.

Bone is not only essential for locomotion, support, and protection but crucial for many aspects of health. Specifically, bone is a calcium/phosphorous reservoir, is integral to glucose metabolism, houses the hematopoietic system and cross-talks with renal and reproductive systems. During development, ossification of the entire post-cranial, endochondral skeleton throughout ontogeny, growth and evolution relies entirely on chondrocytes and the specific extracellular matrix (ECM) they secrete. The continuous transition of cartilage to bone in this endochondral ossification (EO) process is the source of all longitudinal bone growth. A distinct osteoblast cell type is responsible for shaping and maintaining bone formation upon this cartilage ECM template. Of course, some bones arise intramembranously, wherein embryonic mesenchymal cell condensations differentiate directly into osteoprogenitor cells and later into osteoblasts (OB), which secrete an unmineralized osteoid bone matrix around blood vessels. The compact layer of mesenchymal cells surrounding the skeletal element becomes the periosteum; OB on its inner surface deposit osteoid to form layers of bone (Hall, 2005).

**Figure 1:**
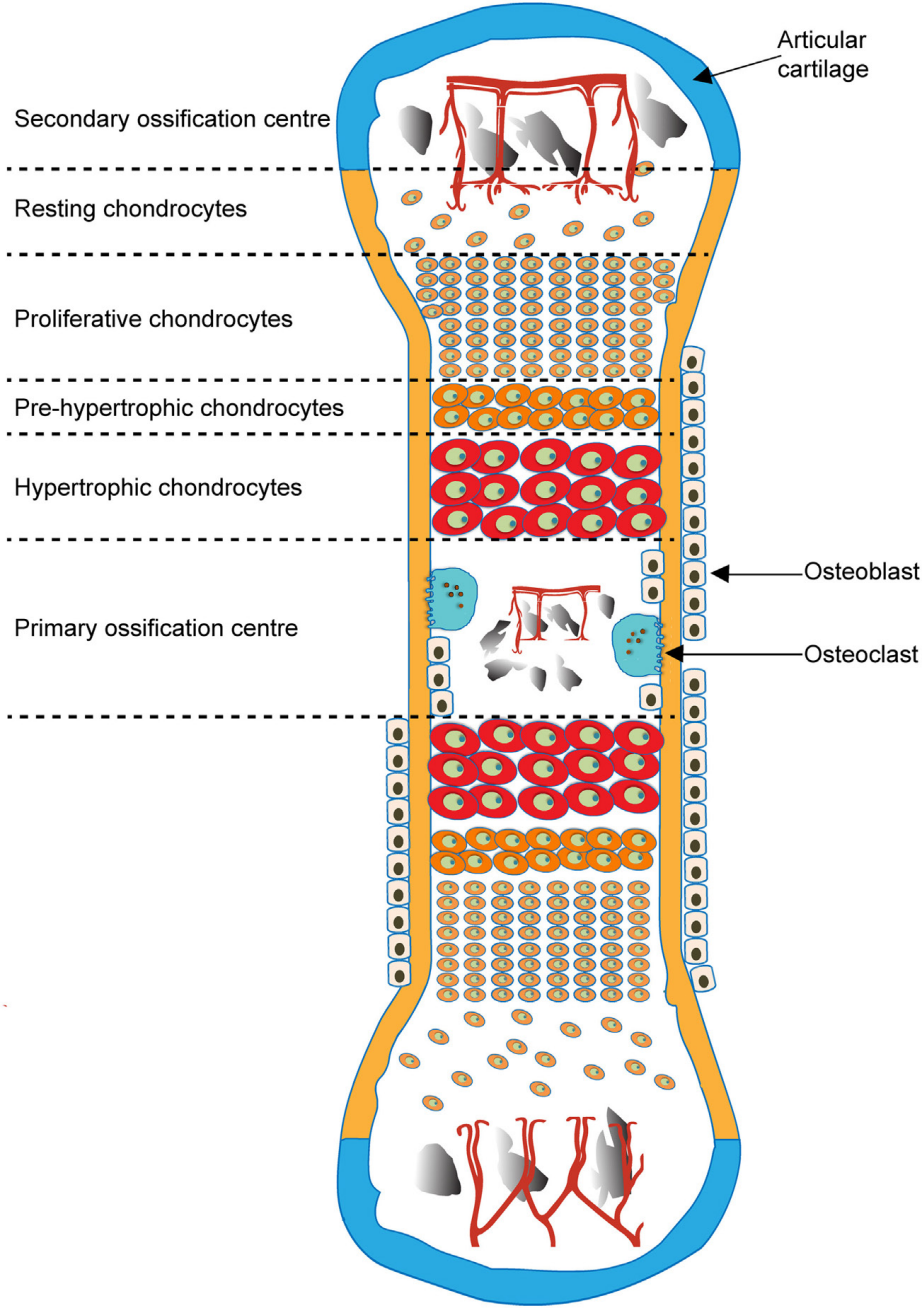
Schematic of endochondral ossification and formation of primary and secondary ossification centers.

Endochondral ossification is initiated by migration of mesenchymal cells to form pre-cartilage condensations, which then undergo differentiation into chondrocytes that secrete ECM rich in aggrecan (Acan) and collagen type II. Calcification of this cartilaginous ECM in this primary ossification center is rapidly followed by its partial replacement and use as a template upon which bone is deposited by OB. This process involves the “propulsion” of the growth plate toward the epiphysis by a burst of proliferation, column formation followed by differentiation into pre-, early- and late, “terminally” differentiated, hypertrophic chondrocytes, associated with an increase in cell volume and deposition of their transient, calcified cartilage matrix (Ballock and O’keefe, 2003; Provot and Schipani, 2005; Mackie et al., 2008). The growing cartilage is invaded by blood vessels (Zelzer et al., 2002), leading to infiltration of bone-resorbing osteoclasts and OB, and ultimately cartilage matrix resorption and new bone formation ensue. Resorption at the chondro-osseous interface leaves calcified cartilage spicules on which OB deposit osteoid to form the primary trabecular bone spongiosa. EO spreads longitudinally from this primary center toward the bone ends and eventually a secondary ossification center forms, retaining the cartilaginous growth plate between each epiphysis and the primary ossification center (Figure 1) (Mackie et al., 2008).

In the context of bone healing, these EO processes are critically important in bridging the bone defect with a cartilage template that is later replaced by bone. Impaired bone healing is characterized by failure to undergo full EO in the cartilage tissue that is formed. In OA, in contrast, there is a failure to retain the stability of the articular cartilage at the joint’s surface, which itself is required to sustain the mechanical environment required for healthy joint motion. Instead, it exhibits undesirable EO-like characteristics during which articular cartilage becomes vascularized, mineralized, and eventually replaced by bone (Kawaguchi, 2008). This EO is particularly evident at the joint margins where osteophytes—bony outgrowths—are formed (Pottenger et al., 1990; Boegård et al., 1998). It may also characterize the bone-marrow lesions now established as another OA hallmark (Felson et al., 2001; Kuttapitiya et al., 2017). Despite clear intimacy of this chondro-osseous interplay in growth, bone healing and OA, the mechanisms reinforcing the chondrocyte: osteoblast inter-relationship are incompletely defined.

An example, relates to the fate of the terminally differentiated chondrocytes. Historically, numerous studies have provided evidence that these hypertrophic chondrocytes undergo apoptosis as their final inevitable fate (Farnum and Wilsman, 1987, 1989; Gibson et al., 1995; Zenmyo et al., 1996; Aizawa et al., 1997; Gibson, 1998). The molecular mechanisms are not fully elucidated but molecular apoptotic signatures; activation of caspases and decreased expression of anti-apoptotic factor Bcl-2, have been reported in hypertrophic chondrocytes (Amling et al., 1997; Adams and Shapiro, 2002). Until recently, consensus was also that all OB originated from invading, periosteal-derived osteoprogenitor cells (Colnot et al., 2004; Maes et al., 2010). Remarkably, hypertrophic chondrocytes lack morphological characteristics of apoptosis; which challenges earlier reports (Emons et al., 2009; Carames et al., 2010) as it would be expected that cell chromatin condensation and nuclear fragmentation would readily be visible. Instead, the presence of autophagic vacuoles and expression of autophagy-regulating genes by growth plate chondrocytes suggest that they instead undergo processes resembling autophagy (Roach and Clarke, 2000; Shapiro et al., 2005).

**Figure 2:**
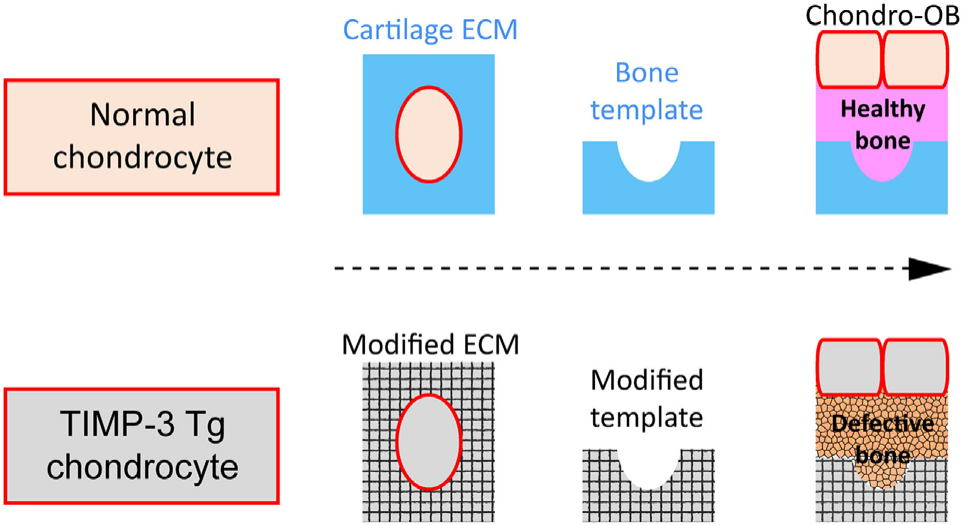
Schematic of hypothesis. **(A)** Normal cartilage extracellular matrix (ECM) (blue) as template for successful (pink) bone formation by osteoblasts (OB) derived either from chondrocytes (Chondro-OB, beige, red outline) or from bone-marrow progeny (BM-OB, green); **(B)** Modified ECM (gray texturized) produced by TIMP3 Tg chondrocytes (gray, red outline) as template for defective bone formation by Chondro-OB (gray, red outline).

It is clear nonetheless that these EO-related events are regulated by paracrine and endocrine signals (Day et al., 2005) including ECM constituents and ECM-modifying enzymes [matrix metalloproteinases (MMPs)] and their regulators (tissue inhibitors of MMPs; TIMPs) (Murphy and Nagase, 2008). Herein, we will (i) explore recent evidence that revisits the idea that hypertrophic chondrocytes may transdifferentiate into a specific sub-population of osteoblast cells and (ii) share data suggesting that osteoblast behavior in this sub-population relies partly upon a direct contribution from the cartilage ECM composition. There are multiple lines of evidence, including our own, showing that local, selective alterations in cartilage ECM remodeling indelibly alter bone quality. This would be consistent with the hypothesis that osteoblast behavior in long bones is regulated by a combination of their lineage origins and the epigenetic effects of chondrocyte-derived ECM which they encounter during their recruitment (Figure 2).

## Emergence of a Chondro-Osseous Cellular Continuum

Endochondral ossification is predominantly understood through its function in long bone development, where mesenchymal-derived chondrocytes undergo a multistep process. These steps correspond to phases in which a precursor pool is maintained, an expansion in cell number, a halt upon division, entry into differentiation and matrix production and finally a dramatic increase in size occur (Tsang et al., 2015). Hypertrophic chondrocytes are often characterized by expression of MMP-13, type X collagen and vascular endothelial growth factor (VEGF) that are hallmarks of their terminal maturation status. It has been assumed that these hallmarks precede their apoptotic death (Farnum and Wilsman, 1987, 1989; Gibson et al., 1995; Zenmyo et al., 1996; Aizawa et al., 1997; Amling et al., 1997; Gibson, 1998; Adams and Shapiro, 2002), which heralds the replacement of cartilage by bone that is initiated by OB and accompanied by vascular invasion of the ECM (Ortega et al., 2004).

An alternative fate and a question that was originally posed more than a century ago (Vander Stricht, 1890; Brachet, 1893) was: can hypertrophic chondrocytes “recycle,” become OB and, therefore, contribute to the osteogenic lineage? This alternative theory has for a long time been a source of controversy. Initially, Dodds and Cameron used histological analysis in rats with rickets to suggest that cartilage cells undergo a form of differentiation that facilitates their eventual transition into osteocytes (Dodds and Cameron, 1939). Consistent with these suggestions, recent studies using a range of *in vivo* and *in vitro* strategies have demonstrated that hypertrophic chondrocytes can traverse this chondro-osseous interface to persist as OB and endorse their ultimate osteocytogenesis to assume a master bone-regulatory function. These studies indicate that newly transdifferentiated OB populate both trabecular and mature cortical bone.

A number of studies explored this alternative fate further; Yoshioka and Yagi (1988) found hypertrophic chondrocytes within primary spongiosa of the rat mandibular condylar cartilage. In addition, Roach and others (Roach, 1992; Roach et al., 1995; Erenpreisa and Roach, 1996) reported asymmetric hypertrophic chondrocyte division in chick bones with one daughter cell transdifferentiating into an osteoblast and the other experiencing apoptosis. Several other studies reported, even in the absence of direct evidence for transdifferentiation, that hypertrophic chondrocytes and OB share many similarities including an osteogenic-specific gene expression profile; both express alkaline phosphatase, osteonectin, osteocalcin, osteopontin, and bone sialoprotein; suggesting common origins (Silbermann et al., 1983; Moskalewski and Malejczyk, 1989; Thesingh et al., 1991; Cancedda et al., 1992; Roach, 1992; Galotto et al., 1994; Roach et al., 1995; Gerstenfeld and Shapiro, 1996; Riminucci et al., 1998; Enishi et al., 2014).

Survival of hypertrophic chondrocytes during EO was found in pathology; chondrocytes in fracture repair calluses exhibited transdifferentiation to OB contributing to ossification. Convincing experimental evidence for a continuous chondrocyte-to-osteoblast lineage also came from use of a cell specific, tamoxifen inducible genetic recombination approach, where chondrocytes from the cartilage anlagen/growth plate were found as OB forming new bone, contributing to fracture repair (Zhou et al., 2014). This conclusion was supported by results from Yang et al., who used Col10a1 driven Cre/loxP to specifically target and tag hypertrophic chondrocytes *in vivo* to report that a hypertrophic chondrocyte sub-population expressing Col10a1 transform into Col1a1-expressing OB and mature osteocytes that express SCLEROSTIN, pre-/post-natally and during fracture repair (Yang et al., 2014). These OB, like those derived directly from osteoblast progenitors, potentially participate in other pathological states including OA. It is reasonable to postulate that any inherent chondrocytic defect could ultimately impact the behavior of their resulting bone cells and thus, that any gene expressed by chondrocytes influences this sub-population of OB ultimately affecting osteogenesis. Jing et al. reported that targeted cartilage-specific cell lineage-tracing leads not only to severe defects in chondrogenesis but complete cessation of EO, with absence of cartilage “remnants” in the subchondral bone (Jing et al., 2015). More importantly, bone phenotypes should now be scrutinized for mutations that also affect cartilage (Bahney et al., 2014).

This notion was further supported by an exploration of stem cell behaviors in endochondral bone healing, which revealed activation of the pluripotent transcription factor, Oct4A—a stem cell marker with proposed cell reprogramming roles—in vascularizing tissues and hypertrophic chondrocytes (Bahney et al., 2014). Despite poor clarity regarding Oct4A function, it appears that chondrocytes dedifferentiate by mechanisms resembling those described for induced pluripotent cells, to regain progenitor capabilities. This would be consistent with mechanism described by Song and Tuan (2004). Elucidation of the cellular reprogramming mechanism(s) that direct this transition from cartilage to bone is, therefore, an obvious target. However, the substantial overlap that exists between markers of hypertrophic chondrocytes and OB represent a significant hurdle in such elucidation (Lian et al., 1993; Stafford et al., 1994; Hughes et al., 1995; Gerstenfeld and Shapiro, 1996). These difficulties in discerning the reprogramming mechanisms are not altogether surprising, since chondrocytes and OB are known to share common osteochondral progenitor origins. More recently, Wang et al. found that SHP2, a cytoplasmic protein tyrosine phosphatase, modulates chondrocyte-to-osteoblast differentiation and that SHP2 deletion exerts a negative skeletal impact (Wang et al., 2017).

Ono et al. revealed that cells expressing Cre-recombinases driven by the collagen II (Col2) promoter/enhancer, and their descendants, contributed to osteoblast progenitors before Runx2 expression; using inducer Col2-creER, Sox9-creER, and Acan-creER approaches these authors reported that early postnatal cells progressively contribute to multiple mesenchymal lineages (Ono et al., 2014). Moreover, Enishi et al. reported that chondrocytes switch to osteoblast-like cells after vascular invasion (Enishi et al., 2014). More recently, Hu et al. described a spatially dependent phenotypic overlap between hypertrophic chondrocytes and OB at the chondro-osseous border in fracture callus, where the former activate expression of the pluripotency factors Sox2, Oct4 (Pou5f1), and Nanog (Hu et al., 2017). These studies also demonstrated that endothelial cell conditioned medium upregulates these genes in *ex vivo* fracture cultures, supporting histological evidence that transdifferentiation occurs adjacent to the vasculature. Moreover, Park et al. (2015) employed BAC-Col10-Cre deleter mice to activate ROSA26 LacZ and ROSA26 YFP reporter genes specifically in hypertrophic chondrocytes (Gebhard et al., 2008). They found that BAC-Col10-Cre;ROSA26 driven LacZ and YFP expression was restricted to hypertrophic chondrocytes before primary ossification center formation, but that substantial osteoblast numbers were positive for β-gal or YFP after onset of bone-marrow formation, leading to the proposal that these OB originated from Col10a1-expressing chondrocytes. This work further revealed the existence of a population of hypertrophic chondrocytes close to the chondro-osseous junction that express stem cell and osteoblast markers (Park et al., 2015). In addition, Jing et al. (2015), using cartilage-specific cell lineage–tracing in mice containing ROSA 26tdTomato, 2.3 Col1GFP, and Acan CreERT2 or Col10-Cre reported that hypertrophic chondrocytes collectively expressed high levels of the anti-apoptotic protein, BCL2. In addition, alkaline phosphatase immunoreactivity was strong in hypertrophic chondrocytes, and BrdU data showed that some hypertrophic chondrocytes undergo cell division. Thus, hypertrophic chondrocytes resemble metabolically active OB rather than simply being inert, metabolically inactive cells, waiting to undergo apoptosis (Jing et al., 2015). These novel findings suggest that, at least, some OB originate from a source distinct from the periosteal-derived osteoprogenitors. Might OB with distinct origins vary in their behavior and activity?

We previously investigated whether OB with divergent origins from different bone types exhibit divergence in their behavior, by comparing growth, differentiation, and angiogenic potential of OB derived from structurally distinct subchondral, trabecular, and cortical bone from a singular skeletal vicinity (Clarkin and Olsen, 2010; Shah et al., 2015). We found that OB from trabecular bone showed slower proliferation, but higher RUNX2, SP7 and BSP-II mRNA levels, TNAP mRNA and protein activity, and lower TNFRSF11B:TNFSF11 mRNA ratios compared to subchondral and cortical bone. In contrast, subchondral OB showed higher VEGF-A mRNA and protein release, implying more intimate vascular relationships (Shah et al., 2015). These findings are consistent with data showing that the response of trabecular, cortical, and subchondral bone to *in vivo* challenges is not always identical (Lavigne et al., 2005; Wade-Gueye et al., 2012), with distinct transcriptional signatures in OB from long bone and calvaria (Akintoye et al., 2006; Rawlinson et al., 2009) and with OB displaying differing gene transcription depending on anatomical location (Candeliere et al., 2001). These data raise the possibility that osteoblast behaviors reflect progenitor origins related to the crossing of a chondro-osseous continuum.

The clearly strong relationships between cells of the growth plate, OB and the vasculature have been dealt with elegantly elsewhere (Maes, 2013). With particular relevance to the concepts shared herein, it is important that we highlight changes to ECM-modifying proteins have been shown to modify vascularization pre- and post-natally. Previous studies have shown that disruption of matrix remodeling *via* modification in MMPs leads to delayed vascularization, inefficient bone repair, and significant alteration in bone mass and architecture (Vu et al., 1998; Holmbeck et al., 1999; Zhou et al., 2000; Colnot et al., 2003; Stickens et al., 2004; Behonick et al., 2007; Lieu et al., 2011). Does this show that the behavior of OB derived from chondrocytes is determined by the cartilage ECM they encounter?

## Do Modifications in the Chondro-Osseous ECM Epigenetically Modify Osteoblast Behavior?

The ECM is a powerful driver of cell behavior. ECM–cell relationships are tightly conserved across evolution, highly dynamic and extend beyond mechanical control of cell fate. Defining how ECM “messages” guide cell performance provides translational impact for stem cell biologists, tissue engineers and biologists to transform approaches to regenerate, repair and remodel connective tissue across pathophysiological contexts. The ECM of the growth plate represents an alternative stimulus to regulate the behavior of cells crossing this chondro-osseous continuum, demonstrating the full intimacy of chondrocyte-osteoblast interplay.

Bone quality and functional integrity relies on interplay between resident cells and the cartilage ECM template. Evidence for this is strong; local control of cartilage ECM remodeling has down-stream effects on bone formation, with mice deficient in the MMPs and TIMP3 having severely disrupted growth plate ECM and bone defects (Vu et al., 1998; Holmbeck et al., 1999; Zhou et al., 2000; Stickens et al., 2004; Javaheri et al., 2016). Does this show that osteoblast behavior is determined by the cartilage ECM they encounter? It seems likely as expression of factors crucial to cartilage signaling (Indian hedgehog, patched and parathyroid hormone-related peptide) are not modified (Karaplis et al., 1994; Marigo et al., 1996; Vortkamp et al., 1996; Maeda et al., 2007). Furthermore, selective chondrocyte-specific Sox9-Cre mediated loss of disintegrin and metallopeptidase domain containing enzyme (ADAM17) leads to bone defects without modifying intrinsic proliferation of resident chondrocytes. Thus, chondrocyte ECM, not solely humoral factors or growth plate dynamics may guide bone mass, architecture, and function (Javaheri et al., 2016).

To test this hypothesis, we created mice with severely modified cartilage ECM remodeling by targeting TIMP3, a member of the metzincin family of MMP inhibitors that inhibits a wide spectrum of ECM-modifying enzymes. Our work in TIMP3-deficient mice and mice overexpressing TIMP3 specifically in chondrocytes, *via* a collagen type II promoter/enhancer (TIMP3 Tg) reveals (i) transient long bone shortening that is restored before adulthood; (ii) diminished bone mass with defective architecture and lower fracture resistance; (iii) restriction of TIMP3 Tg impact to only endochondral bones; and (iv) deficiencies in osteoblast proliferation and differentiation that persists *in vitro*. Our data show that bone formation relies partly upon the cartilage ECM encountered during osteoblast recruitment (Javaheri et al., 2016; Poulet et al., 2016). Our work also shows that aggrecanase inhibition increases and collagenase inhibition decreases bone mass, suggesting that balance in their activities is crucial; however, the identities of the growth plate ECM components that underpin these divergent phenotypes remains obscure.

While our findings confirm the existence of a cellular chondro-osseous continuum, they also crucially pinpoint how behavior of cells with divergent origins is controlled *in vivo* by the ECM they encounter. Since growth plate calcified cartilage serves as a template not only for bone formation but also osteoclast-mediated resorption, this implies that osteoclast behavior may also be modified in TIMP3 Tg mice to subsequently influence bone formation. However, our pilot studies suggest this is unlikely as we find no significant changes in osteoclast numbers in TIMP3 Tg mouse bone (Javaheri et al., 2016). In contrast, we find that OB derived from these TIMP3 Tg mice have defective differentiation even upon *in vitro* isolation, supporting the likelihood that “imprinted” epigenetic modifications are contingent upon the ECM the OB encounter (Poulet et al., 2016). The precise nature of these epigenetic effects of chondrocyte-derived ECM remains unexplored. Nonetheless, multiple lines of evidence, including ours, show that local alterations in cartilage ECM remodeling indelibly alter bone quality, leading us to hypothesize that osteoblast behavior in long bones is regulated by a combination of their lineage origins and the epigenetic effects of chondrocyte-derived ECM they encounter during recruitment.

## Conclusion

Recent evidence challenges a long-held paradigm regarding EO, indicating that chondrogenesis and osteogenesis are more closely linked than appreciated and, that cells derived originally from the cartilage niche, as opposed to solely osteoprogenitors, transform into bone-forming OB and ultimately osteocytes. We offer a fundamental extension to this paradigm proposing that these OB are epigenetically influenced by ECM encountered during this transition. We need to ascertain how hypertrophic chondrocyte transdifferentiation fate is controlled and which specific ECM factors regulate this transition to impact bone quality and integrity. This new concept challenges us to re-evaluate cartilage and bone stages in EO, such that we treat them as a continuum rather than entirely independent. It also prompts us to revisit EO as an “evolutionary” shift in which cartilage might provide new cells that can instead form bone rather than simply as a means of lengthening skeletal elements. As impaired bone healing and OA both involve EO it is conceivable that tissue engineering approaches might exploit these developmental principles, embracing the newly established cellular chondro-osseous continuum to better control cell fate decisions.

## Author Contributions

Conceived and designed the idea: BJ, GG, and AP. Wrote the mini review: BJ, SC-S, IK, GG, and AP. Revision and finalizing the mini review: BJ, GG, and AP.

## Conflict of Interest Statement

The authors declare that all financial, commercial or other relationships that might be perceived by the academic community as representing a potential conflict of interest are disclosed.
